# LRez: a C++ API and toolkit for analyzing and managing Linked-Reads data

**DOI:** 10.1093/bioadv/vbab022

**Published:** 2021-09-25

**Authors:** Pierre Morisse, Claire Lemaitre, Fabrice Legeai

**Affiliations:** 1 Univ Rennes, Inria, CNRS, IRISA, Rennes 35000, France; 2 IGEPP, INRAE, Institut Agro, Univ Rennes, Rennes 35000, France

## Abstract

**Motivation:**

Linked-Reads technologies combine both the high quality and low cost of short-reads sequencing and long-range information, through the use of barcodes tagging reads which originate from a common long DNA molecule. This technology has been employed in a broad range of applications including genome assembly, phasing and scaffolding, as well as structural variant calling. However, to date, no tool or API dedicated to the manipulation of Linked-Reads data exist.

**Results:**

We introduce LRez, a C++ API and toolkit that allows easy management of Linked-Reads data. LRez includes various functionalities, for computing numbers of common barcodes between genomic regions, extracting barcodes from BAM files, as well as indexing and querying BAM, FASTQ and gzipped FASTQ files to quickly fetch all reads or alignments containing a given barcode. LRez is compatible with a wide range of Linked-Reads sequencing technologies, and can thus be used in any tool or pipeline requiring barcode processing or indexing, in order to improve their performances.

**Availability and implementation:**

LRez is implemented in C++, supported on Unix-based platforms and available under AGPL-3.0 License at https://github.com/morispi/LRez, and as a bioconda module.

**Supplementary information:**

Supplementary data are available at *Bioinformatics Advances* online.

## 1 Introduction

Linked-Reads technologies, pioneered by 10× Genomics (Zheng *et al.*, 2016), partition and tag high-molecular-weight DNA molecules with a barcode using a microfluidic device prior to classical short-read sequencing. This way, all the sequenced reads that come from a common molecule contain an identical barcode, thus offering additional data for downstream processing, compared to classical short reads. As a result, Linked-Reads manage to combine the high quality of the short-reads and long-range information, which can be inferred by identifying distant reads belonging to the same DNA molecule with the help of the barcodes. Although 10× Genomics has recently discontinued their Linked-Reads products, large volumes of data were produced and still need to be analyzed. Moreover, three other Linked-Reads technologies have been developed and commercialized in the last 2 years, namely TELL-seq (Chen *et al.*, 2020), stLFR (Wang *et al.*, 2019) and the open protocol Haplotagging (Meier *et al.*, 2021). They have already produced many such data and will likely increase their throughput in the future. For instance, the lower cost of Haplotagging, with respect to long-read technologies is very attractive, especially for large-population resequencing projects.

**Fig. 1.**
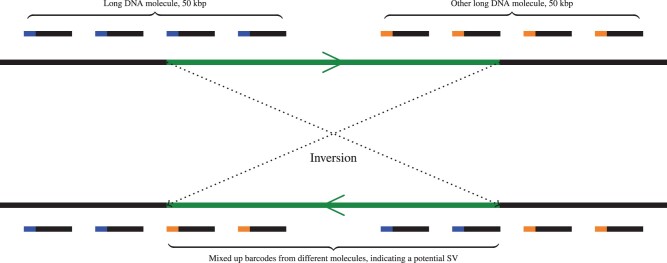
Illustration of the barcodes functionalities and interest. Top: Barcoded reads from two different long DNA molecules aligned to a reference genome, underlining the interest of the barcode (colored segment at the beginning of the reads) for long-range information retrieval. Bottom: Example of the barcodes’ benefits in the context of structural variant calling. If the green segment of the reference genome undergoes an inversion, the barcodes from the two different long DNA molecules get mixed up together, and can serve as an indicator allowing to retrieve the structural variant location

Linked-Reads have already been efficiently employed in various applications, such as structural variant calling (Spies *et al.*, 2017), and also genome assembly (Mostovoy *et al.*, 2016), phasing (Zheng *et al.*, 2016) and scaffolding (Yeo *et al.*, 2018). To benefit from Linked-Reads data, most methods first map the reads against a reference genome, and then rely on the analysis of the barcode contents of genomic regions, often requiring to fetch all reads or alignments with a given barcode. Figure 1 illustrates the overall functionality of the barcodes, as well as their usefulness in the context of structural variant calling. However, despite the fact that various tools and libraries, such as SAMtools (Wysoker *et al.*, 2009) and BamTools (Barnett *et al.*, 2011), are available for processing BAM files, to the best of our knowledge, no such tool currently exists for managing Linked-Reads barcode and allowing features, such as indexing, querying and comparisons of barcode contents. LRez aims to address this issue, by providing a complete and easy to use API and suite of tools that are compatible with various Linked-Reads sequencing technologies.

To emphasize the usefulness of LRez, the API is already used in the structural variant calling tool LEVIATHAN (Morisse *et al.*, 2021), where its indexing features are used to identify pairs of distant genomic regions sharing a higher than expected number of barcodes, making this the fastest and most memory-efficient tool in the state-of-the-art. Furthermore, the FASTQ indexing and querying features of the LRez toolkit are currently used in the gap-filling pipeline MTG-Link (https://github.com/anne-gcd/MTG-Link), to efficiently retrieve read sequences, selected based on their barcodes, for local *de novo* assembly.

## 2 Features and methods

### 2.1 LRez toolkit

The LRez toolkit provides end-users with a suite of command-line programs for indexing and manipulating Linked-Reads barcodes, in BAM, FASTQ and gzipped FASTQ files, where the barcodes are reported using the BX:Z tag. This tag is classically used by all Linked-Reads sequencing technologies to report barcode information, in both FASTQ and BAM files. In case, the input data are not formatted as so, some scripts are provided by LRez, and external programs, such as Long Ranger basic can also be used to properly format the data. Table 1 provides a summary of currently available features of LRez, and we further describe some of these features below. In all features, barcode extraction from BAM files is performed using the BamTools library (Barnett *et al.*, 2011).

**Table 1.** LRez command-line toolkit features

Command	Description
compare	Compute the number of common barcodes between pairs of regions or between pairs of contig ends
extract	Extract the barcodes from a given region of a BAM file
stats	Compute barcode statistics from a BAM file
index bam	Index the BAM offsets or genomic positions of the barcodes contained in a BAM file
index fastq	Index by barcode the offsets of the sequences contained in a FASTQ or gzipped FASTQ file
query bam	Query the index to retrieve alignments in a BAM file given a barcode or list of barcodes
query fastq	Query the index to retrieve sequences in a FASTQ or gzipped FASTQ file given a barcode or list of barcodes

#### 2.1.1 Index and query BAM and FASTQ files

**Fig. 2.**
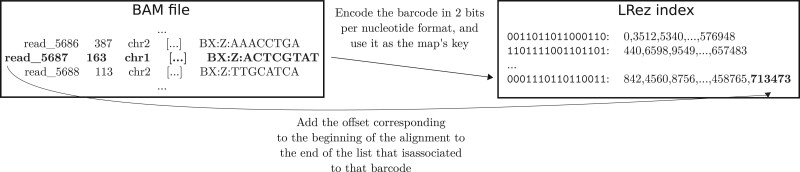
Procedure for building an offset index from a BAM file. The index is represented as a map, where the keys are the barcodes in 2 bits per nucleotide representation, and where the values are lists of alignments offsets. To build the index, the BAM file is browsed sequentially. For each alignment, the associated barcode is encoded in 2 bits per nucleotide representation, to retrieve the key. The offset corresponding to the alignment is then added to the end of the offsets list of the barcode

The index bam command can be used to build two types of indexes. In each case, the index is represented as a map, where the key is the barcode, recorded using 2 bits per nucleotide to mitigate memory consumption, and where the value is a list of elements depending on the index type. The first type of index records the offsets of the barcodes in the BAM file, allowing to quickly retrieve all alignments involving reads with a given barcode. The second type of index records the genomic positions of the alignments of reads in which the barcode appears. It can then be queried, for instance, to quickly retrieve the chromosomes on which a given barcoded molecule appears. The indexing procedure for the first kind of index is illustrated in Figure 2. Moreover, the construction of both types of indexes supports additional parameters allowing to only consider barcodes from primary alignments and/or having a mapping quality higher than a user-specified value. These options allow to restrict the barcodes of interest to the most valuable ones for further analysis, and thus reduce memory footprint and querying time. In the same fashion, the index fastq subcommand, allows to index FASTQ and gzipped FASTQ files, by storing all the offset positions of the reads, allowing to rapidly extract reads given any barcode.

#### 2.1.2 *Compare*

The compare subcommand computes the number of common barcodes between pairs of regions. Two types of comparison are implemented. First, when only a few regions need to be compared, it computes common barcodes counts between all possible pairs of regions without relying on the index. It does so by performing greedy extraction and comparison of the barcode contents of each region. The second feature, motivated by scaffolding applications, compares the ends of a given contig of interest against all other contigs’ ends. This time, the index is used. The barcodes from the extremities of the contig of interest are extracted, and the index is queried in order to retrieve other contigs’ ends in which these barcodes appear, and compute the numbers of common barcodes on-the-fly. Both functionalities of the compare are illustrated in Supplementary Materials Section S1 (*LRez compare*) and Figure S1.

#### 2.1.3 *Extract*

The extract subcommand allows extracting barcodes from a BAM file, either for a specified region of the reference genome or the whole BAM file. When a region is specified, LRez simply jumps to the region of the BAM file containing the alignments of interest, and extracts the barcodes. The extract subcommand also has an additional parameter, allowing to include duplicate barcodes during extraction, which can be useful for applications relying on barcodes counts.

#### 2.1.4 *Stats*

The stats subcommand computes barcode statistics from a BAM file. In terms of global statistics, it reports the total number of barcodes and mapped reads. It also reports more specific statistics describing the distributions of the number of reads per barcode, the number of barcodes per region and the number of common barcode between adjacent regions. For these more detailed statistics, a given number of regions are picked randomly along the genome and are analyzed, with the size and number of regions to consider that can be user-specified. The 1st quantile, the median, as well as the 3rd quantile of the observed distributions are then reported.

### 2.2 LRez API

The LRez API provides C++ programmers with tools allowing efficiently analyze and manage Linked-Reads data. It is compiled as a shared library, helping its integration to external projects. Moreover, all functionalities are implemented in a thread-safe fashion. Available modules include IndexManagementBam and IndexManagementFastq, to build indexes from BAM and FASTQ files, AlignmentsRetrieval and ReadsRetrieval, to query the indexes and retrieve alignments or reads tagged with a given barcode, BarcodesExtraction and BarcodesComparison, to extract barcodes from given regions of a BAM file, and compute the number of common barcodes between pairs of regions and finally computeStats, to extract statistics from a BAM file. Guidelines on how to use the API and code examples are provided on the GitHub project. An example is also provided in the Supplementary Materials Section S7 (*Example of API usage*).

## 3 Results

We tested the index bam and query bam subcommands on multiple files from various organisms and sequencing technologies. The datasets are described in details in Supplementary Materials Section S2 (*Linked-Reads datasets*). All experiments were performed on a cluster node equipped with 150 GB of RAM using a single thread, on a 2.3 GHz CPU. Indexing results are reported in Table 2, and querying results are reported in Table 3. We compared the querying results with two naive methods without a barcode-based indexing of the reads. The first naive method we chose was to use the grep -f command, with the list of query barcodes as a parameter, piped to the output of the samtools view command. The second approach, which is less naive but still index-free, is to use a Python script performing a single scan through the input file, with the list of query barcodes stored in a dictionary. We report the total runtime for performing a 1000 barcodes query, as well as the average runtime per queried barcode. Additionally, for LRez, we report the runtime of the indexing and querying steps combined, as well as the runtime of the querying step alone. We also provide the command lines that were used to run LRez, both for indexing and querying, in Supplementary Materials Section S3 (*Indexing experiments*) and Section S4 (*Querying experiments*).

**Table 2.** LRez runtime and memory consumption for indexing BAM files from different species and sequencing technologies

Dataset	BAM	# Barcodes	Runtime	Runtime	RAM	Disk
	size (GB)		(1 thread)	(8 threads)	(MB)	(MB)
10× Genomics (*H. sapiens*)	61	609 058	52 min	31 min	9320	15 062
Haplotagging (*H. erato*)	70	36 645 651	1 h 09 min	42 min	10 751	10 125
stLFR (*H. sapiens*)	206	38 779 362	3 h 06 min	1 h 50 min	26 769	34 256
TELL-Seq (*E. coli*)	1	634 133	1 min	35 s	293	340

The disk column corresponds to the disk size occupied by the serialized index.

**Table 3.** Runtimes for performing a query of 1000 barcodes on BAM files, using LRez and naive approaches based on grep + SAMtools and single scans through the whole files using a Python script

	Overall runtime				Runtime per query		
Dataset	grep^a^	Python	LRez	LRez	grep^a^	Python^a^	LRez^b^
			(index + query)	(query)			
10× Genomics (*H.sapiens*)	13 h	46 min	1 h 02 min	10 min	1.7 min	2.8 s	290 ms
Haplotagging (*H.erato*)	10 h	42 min	1 h 14 min	5 min	36 s	2.5 s	11 ms
stLFR (*H.sapiens*)	12 h	2 h 26 min	3 h 14 min	8 min	43 s	2.5 s	15 ms
TELL-Seq (*E.coli*)	31 s	42 s	1 min	7 s	31 ms	42 ms	4 ms

For LRez, we report the overall runtime, including both the indexing and querying steps, as well as the runtime of the querying step alone. Additionally, we also report the average runtime per query, both for the naive approaches and LRez.

a Times per query were obtained by dividing the overall runtime by the number of queries. In practice, these approaches would still require a whole scan through the file for a single query, and actual runtime for querying a single barcode would be larger.

b Times per query do not take into account indexing time.

These experiments show that, on a 61 GB 10× Genomics BAM file from *Homosapiens*, indexing took an hour, and resulted in an index occupying 9 GB of RAM. With this index, querying time per barcode reached an average of 290 ms. In comparison, using naive approaches without a barcode-based index, querying time per barcode reached 1.7 min with the grep-based approach, and 2.8 s with the Python-based approach. On another 204 GB stLFR BAM file from *H.sapiens*, containing less alignments per barcode, indexing required 26 GB of RAM and LRez querying time only reached an average of 15 ms, while the naive approaches based on grep and Python required 43 and 2.5 s per query, respectively. Additional indexing and querying experiments on FASTQ files are reported in Supplementary Materials Section S3 (*Indexing experiments*) and Table S1 and Section S4 (*Querying experiments*) and Table S2. Moreover, it is worth noting that, due to the large main memory size available on the cluster node, the index has always held in the main memory for all experiments, and no additional virtual memory was thus required. Additional experiments showed that, when the size of the main memory is not sufficient to store the index, the indexing process cannot terminate and users shall thus prefer using naive index-free approaches in such cases.

When taking into account the indexing time, LRez is slower than the naive Python-based approach for a single query of 1000 barcodes. However, applications usually perform hundreds to thousands of such queries. Supplementary Materials Section S5 (*Runtime depending on the number of queries*) and Figures S2 and S3 show the runtime depending on the number of queries. These results underline the fact that building the index and querying with LRez is more efficient as soon as two or three queries are required on BAM or FASTQ files. On the TELL-Seq *Escherichiacoli* FASTQ file, a larger number of queries are required to see the benefit of LRez, which can be explained by the fact that this file is much smaller compared to the others, and that it is thus much easier for naive methods to scan through it. On gzipped FASTQ files, the number of queries required to see the interest of LRez also raises. This can be explained by the fact that LRez needs to decompress multiple blocks when querying gzipped FASTQ files, and that decompression is more expensive than simply browsing through a regular file.

We also tested the compare subcommand to compute the number of common barcodes between the extremities of the 1000 largest contigs of a highly fragmented *H.numata* assembly (Jay *et al.*, 2021), on an 11 GB 10× Genomics BAM file. After building the index, processing time per contig reached an average of 2 s. Using a single thread, the comparison between all contigs thus required 33 min, while using eight threads, allowed to reduce the runtime to only 4 min. Instructions for running the compare and the extract subcommands are reported in Supplementary Materials Section S6 (*Running other LRez subcommands*).

## 4 Conclusion

LRez provides a toolkit and an easy to use C++ API, allowing to deal with Linked-Reads data from multiple sequencing technologies, and offering various functionalities. We thus believe it might help both end-users and programmers alike, and be easily integrated to external projects, including structural variant discovery, genome scaffolding or phasing. As previously mentioned, the API is already used in the structural variant calling tool LEVIATHAN, while the FASTQ indexing and querying features of the LRez toolkit are currently used in the gap-filling pipeline MTG-Link. LRez can thus be easily integrated to external projects, allowing to perform various frequent Linked-Reads management tasks in an optimized manner. Using LRez would thus allow developers and users to avoid reimplementing these common tasks, and directly rely on our implementation. As Linked-Reads technologies are becoming more diverse and widespread, we believe LRez has the potential to be used in a wide variety of other tools and applications, and that it will prove useful to the whole community, both now and in the future.

## Data availability statement

No new data were generated or analysed in support of this research.

## Funding

This work was supported by the French Agence Nationale de la Recherche [grant number ANR-18-CE02-0019 Supergene].

*Conflict of Interest*: none declared.

## Supplementary Material

vbab022_Supplementary_Data
