# Supplementary material for: LRez: a C++ API and toolkit for analyzing and managing Linked-Reads data
Source: Bioinform Adv. 2021 Sep 25;1(1):vbab022. doi: 10.1093/bioadv/vbab022 (PMC9710615; doi:10.1093/bioadv/vbab022)
Supplement: vbab022_Supplementary_Data [file vbab022_supplementary_data.pdf]

LRez: C++ API and toolkit for analyzing and managing Linked-Reads data

Supplementary Materials

Pierre Morisse[1,*], Claire Lemaitre[1] and Fabrice Legeai[2]

[1]Univ Rennes, Inria, CNRS, IRISA, 35000, Rennes, France
[2] IGEPP, INRAE, Institut Agro, Univ Rennes, 35000, Rennes, France

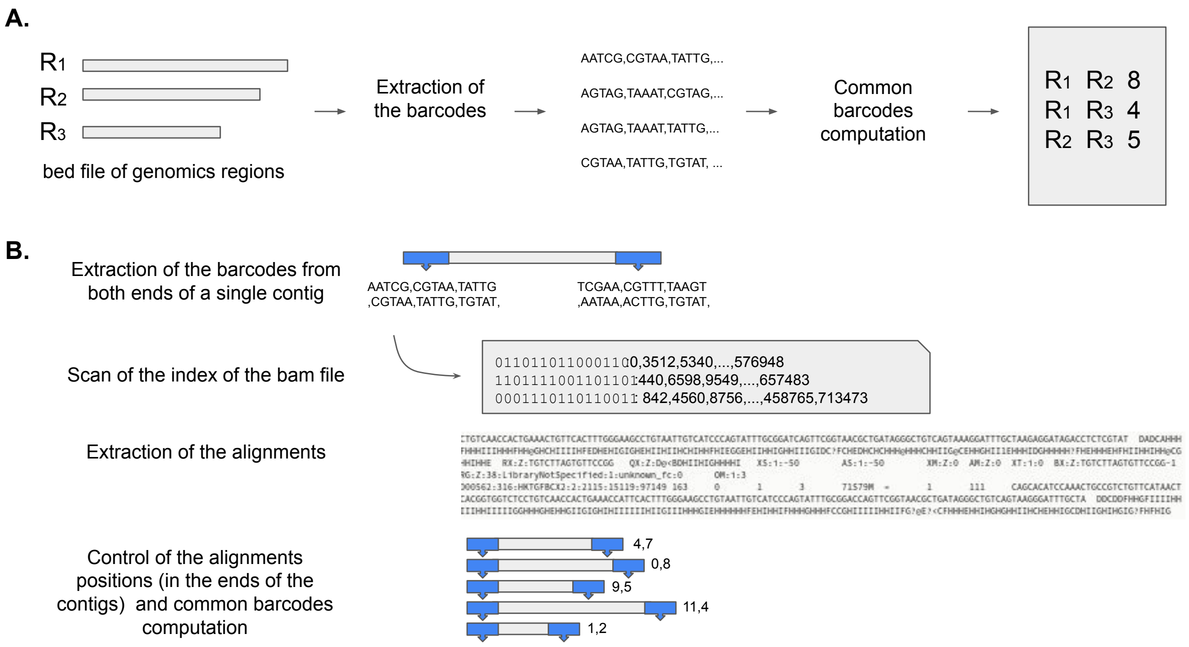

Figure S1: **Schematic overview of the Compare submodule.** Barcodes are presented shorter for clarity sake. A. When a list of regions is provided (as a bed file), it computes the number of common barcodes between all region pairs. B. When a contig is provided, it compares this contig's ends with all the other contigs' ends using the barcode index. The number of common barcodes between the provided contig's extremities and other contigs' extremities is thus computed.

1 LRez compare

A schematic overview of the LRez compare submodule is provided in Supplementary Figure S1. This figure illustrates how LRez behaves when a list of genomic regions to be compared are provided, and when a contig's ends need to be compared to all other contigs' ends.

2 Linked-Reads datasets

LRez was used on various Linked-Reads datasets, from four different Linked-Reads technologies and from different organisms :

- 10x Genomics (*H. sapiens*): sequencing of the HG002 human individual with the 10x Genomics technology. The BAM file is provided by the Genome in a Bottle consortium and was downloaded from the following link: `ftp://ftp-trace.ncbi.nlm.nih.gov/giab/ftp/data/AshkenazimTrio/HG002_NA24385_son/10XGenomics/`
- stLFR (*H. sapiens*): sequencing of the HG002 human individual with the stLFR technology. The BAM/FASTQ files are provided by the Genome in a Bottle consortium and were downloaded from the following link: `https://ftp-trace.ncbi.nlm.nih.gov/giab/ftp/data/AshkenazimTrio/HG002_NA24385_son/stLFR/`
- TELL-Seq (*E. coli*): sequencing with the TELL-seq technology of XX *Escherichia coli* bacteria (genome of ~ 5 Mb). The FASTQ dataset is available from NCBI accession number SRR10584146

[1]. The reference genome is available at the following location: `https://www.ncbi.nlm.nih.gov/nuccore/U00096.2?report=fasta`

- Haplotagging *H. erato*: pooled sequencing with the Haplotagging technology of hundreds of *Heliconius erato* butterfly individuals (genome of ∼ 400 Mb). The BAM file was obtained by personnal communication. It will soon be released under the SRA Project ID PRJNA670070 [2].

2.1 File format conversions

LRez is compatible with these four Linked-Reads technologies. The only pre-requisite is that barcodes must be trimmed from the read sequences, and reported using the `BX:Z` tag in the alignment tags of BAM files and in the reads' headers of FASTQ files. This is a usual pre-processing that is usually performed by the corresponding technologies' tools. Moreover, extracting the barcodes from raw reads as so has no impact on the link information, since aligners are able to report the barcodes in the alignments when they are initially reported in the reads' headers, thus ensuring no information is lost. Moreover, extracting the barcodes allows non-barcode-aware aligners to avoid suprious mappings due to the presence of the barcodes in the reads' sequences. Additionally, if barcode extraction was not performed, the LRez GitHub repository provides scripts in the `utils` folder to perform this pre-processing step.

While the downloaded 10x Genomics and Haplotagging BAM files were already formatted as such, stLFR and TELL-Seq datasets were not publicly distributed in the usual format and needed to be pre-processed.

The stLFR BAM and FASTQ files were pre-processed as follows:

```
python preprocessBAMstLFR.py stLFR_NA24385.sort.rmdup.bam
# output file : stLFR_NA24385.sort.rmdup_barcodes_extracted.bam
python preprocessFASTQstLFR.py stLFR_NA24385_split_read.1.fq
# output file : stLFR_NA24385_split_read.1_barcodes_in_headers.fq.
```

For the TELL-seq datasets, only FASTQ files were provided. We used the following commands to pre-process these files and map the reads and obtain a properly formatted BAM file:

```
# retrieving fastq files from SRA in 2 files
fastq-dump SRR10584146 --split-files
# barcode extraction and header formatting
python preprocessTELL-Seq.py SRR10584146_1.fastq SRR10584146_2.fastq SRR10584146_3.fastq
# output : SRR10584146_1_barcodes_in_headers.fastq
# and SRR10584146_2_barcodes_in_headers.fastq
# read mapping on the reference genome
bwa index Ecoli.fasta
bwa mem -C Ecoli.fasta SRR10584146_1_barcodes_in_headers.fastq \\
    SRR10584146_2_barcodes_in_headers.fastq | samtools view -Sb \\
    | samtools sort > SRR10584146.bam
```

3 Indexing experiments

To assess the indexing functionalities of LRez, we performed experiments on BAM, FASTQ and gzipped FASTQ files. We present the results of these experiments in the following subsections.

3.1 Indexing BAM files

We provide the command lines that were used to run LRez below.

Running LRez We provide the command lines that were used to run LRez below, and also specify the `samtools index` command required to create the `.bai` file associated to the BAM file, when needed. For all these experiments, we used LRez v2.1.1.

- On the 10x Genomics *H. sapiens* dataset:

```
LRez index bam -b NA24385_phased_possorted_bam.bam -f \
    -o NA24385_phased_possorted_bam.bci
```

- On the stLFR *H. sapiens* dataset:

```
samtools index stLFR_NA24385.sort.rmdup_barcodes_extracted.bam
LRez index bam -b stLFR_NA24385.sort.rmdup_barcodes_extracted.bam -f \
    -o stLFR_NA24385.sort.rmdup_barcodes_extracted.bci
```

- On the TELL-Seq *E. coli* dataset:

```
samtools index SRR10584146.bam
LRez index bam -b SRR10584146.bam -f -o SRR10584146.bci
```

3.2 Indexing FASTQ files

Indexing experiments on the aformentioned FASTQ and gzipped FASTQ files are presented in Supplementary Table S1. Additionally, we also provide the command lines that were used to run LRez. Since FASTQ were not available for all the datasets mentioned in Section 2, these experiments were only performed on the stLFR *H. sapiens* dataset and on the TELL-Seq *E. coli* dataset.

**Dataset**	**FASTQ size (GB)**	**# Barcodes**	**Runtime (1 thread)**	**Runtime (8 threads)**	**RAM (MB)**	**Disk (MB)**
stLFR (*H. sapiens*)	295	39,100,215	1 h 21 min	29 min	16,104	13,047
stLFR (*H. sapiens*) gzipped	108	39,100,215	2 h 27 min	1 h 17 min	25,140	13,047
TELL-Seq (*E. coli*)	4.4	786,116	30 sec	10 sec	229	129
TELL-Seq (*E. coli*) gzipped	0.855	786,116	1 min 37 sec	50 sec	344	129

Table S1: LRez runtime and memory consumption for indexing FASTQ and gzipped FASTQ files from different species and sequencing technologies. The disk column corresponds to the disk size occupied by the serialized index.

Running LRez We provide the command lines that were used to run LRez below. For all these experiments, we used LRez v2.1.1.

- On the stLFR *H. sapiens* dataset:

```
LRez index fastq -f stLFR_NA24385_split_read.1_barcodes_in_headers.fq \
    -o stLFR_NA24385_split_read.1_barcodes_in_headers.bci
```

- On the stLFR *H. sapiens* gzipped dataset:

```
LRez index fastq -f stLFR_NA24385_split_read.1_barcodes_in_headers.fq.gz \
    -o stLFR_NA24385_split_read.1_barcodes_in_headers_gzipped.bci -g
```

- On the TELL-Seq *E. coli* dataset:

```
LRez index fastq -f SRR10584146_1_barcodes_in_headers.fastq \
    -o SRR10584146_1_barcodes_in_headers.fastq.bci
```

- On the TELL-Seq *E. coli* gzipped dataset:

```
LRez index fastq -f SRR10584146_1_barcodes_in_headers.fastq.gz \
    -o SRR10584146_1_barcodes_in_headers.fastq_gzipped.bci -g
```

4 Querying experiments

To assess the interest of LRez querying functionalities, we compared them against naive methods to retrieve either alignements from BAM files or reads from FASTQ or gzipped FASTQ files. We present the results of these experiments in the following subsections.

4.1 Querying BAM files

We provide the command lines that were used to run LRez below.

Running LRez We provide below the command lines that were used to run LRez. For each command, the `barcodesList` parameter corresponds to a file containing a list of query barcodes, with one barcode per line. For all these experiments, we used LRez v2.1.1.

- On the 10x Genomics *H. sapiens* dataset:

```
LRez query bam -b NA24385_phased_possorted_bam.bam \
    -i NA24385_phased_possorted_bam.bci -l barcodesList
```

- On the stLFR *H. sapiens* dataset:

```
LRez query bam -b stLFR_NA24385.sort.rmdup_barcodes_extracted.bam \
    -i stLFR_NA24385.sort.rmdup_barcodes_extracted.bci -l barcodesList
```

- On the TELL-Seq *E. coli* dataset:

```
LRez query bam -b SRR10584146.bam -i SRR10584146.bci -l barcodesList
```

4.2 Querying FASTQ files

We performed querying experiments on the four previously indexed datasets. We compared the results with two naive methods without a barcode-based indexing of the reads. The first naive method we chose was to use the `grep -f` command, with the list of query barcodes as a parameter. For gzipped FASTQ files, we used the same approach, piped to the output of the `zcat` command. The second naive approach we used was a Python script performing a single scan through the input file, with the list of query barcodes stored in a dictionary. For gzipped FASTQ files, we once again used the same approach, piped to the output of the `zcat` command. Results of these experiments are reported in Supplementary Table S2. We report the total runtime for performing a thousand queries, as well as the average runtime per query. Additionally, for LRez, we report the runtime of the indexing and querying steps combined, as well as the runtime of the querying step alone. We also provide the command lines that were used to run LRez below.

**Dataset**	**Overall runtime**				**Runtime per query**		
	grep	Python	LRez (index + query)	LRez (query)	grep[1]	Python[1]	LRez[2]
stLFR (*H. sapiens*)	28 hours	37 min	1 h 26 min	5 min	1.7 min	2.2 sec	30 ms
stLFR (*H. sapiens*) gzipped	31 hours	58 min	3 h 14 min	47 min	1.9 min	3.5 sec	860 ms
TELL-Seq (*E. coli*)	3 sec	15 sec	45 sec	2 sec	3 ms	15 ms	1.5 ms
TELL-Seq (*E. coli*) gzipped	21 sec	22 sec	2 min	20 sec	21 ms	22 ms	19 ms

Table S2: Runtimes for performing a query of 1,000 barcodes on FASTQ and gzipped FASTQ files, using LRez and using a naive approach based on grep and on single scans through the whole files using a Python script. For LRez, we report the overall runtime, including both the indexing and querying steps, as well as the runtime of the querying step alone. Additionally, we also report the average runtime per query, both for the naive approaches and LRez. [1] Times per query were obtained by dividing the overall runtime by the number of queries. In practice, these approaches would still require a whole scan through the file for a single query, and actual runtime for querying a single barcode would be larger. [2] Times per query do not take into account indexing time.

Running LRez We provide below the command lines that were used to run LRez. For each command, the `barcodesList` parameter corresponds to a file containing a list of query barcodes, with one barcode per line. For all these experiments, we used LRez v2.1.1.

- On the stLFR *H. sapiens* dataset:

```
LRez query fastq -f stLFR_NA24385_split_read.1_barcodes_in_headers.fq \
    -i stLFR_NA24385_split_read.1_barcodes_in_headers.bci -l barcodesList
```

- On the stLFR *H. sapiens* gzipped dataset:

```
LRez query fastq -f stLFR_NA24385_split_read.1_barcodes_in_headers.fq.gz \
    -i stLFR_NA24385_split_read.1_barcodes_in_headers_gzipped.bci -l barcodesList -g
```

- On the TELL-Seq *E. coli* dataset:

```
LRez query fastq -f SRR10584146_1_barcodes_in_headers.fastq \
    -i SRR10584146_1_barcodes_in_headers.fastq.bci -l barcodesList
```

- On the TELL-Seq *E. coli* gzipped dataset:

```
LRez query fastq -f SRR10584146_1_barcodes_in_headers.fastq.gz \
    -i SRR10584146_1_barcodes_in_headers.fastq_gzipped.bci -l barcodesList -g
```

5 Runtime depending on the number of queries

Usually, applications do not query BAM or FASTQ files to retrieve alignments / reads given a list of barcodes just once, but rather iterate the process many times. For instance, for SV calling applications, some methods divide the reference genome of interest into small regions, and require to query the index with the barcodes of each region. In practice, on a human genome divided into regions of size 10,000 bp, this would result in a total number of 320,000 queries.

To further emphasize the value of LRez, we performed multiple querying experiments using LRez and the naive approaches, on BAM, FASTQ, and gzipped FASTQ files. The results of these experiments are reported in Figure S2 for BAM files, and in Figure S3 for FASTQ and gzipped FASTQ files.

The BAM querying experiments show that, for all datasets except TELL-Seq *E. coli*, which is substantially smaller than the others, indexing the barcodes and using LRez to retrieve alignments is faster than other approaches when at least two queries are required. For the smaller TELL-Seq *E. coli* dataset, LRez becomes more efficient if at least three queries are required.

The FASTQ, and especially the gzipped FASTQ, querying experiments show that given a higher number of queries, indexing the barcodes and using LRez to retrieve reads is still faster than other approaches. The minimum number of queries raises to three for the stLFR *H. sapiens* FASTQ file, and to around 14 for the gzipped version of that same file. On the smaller TELL-Seq *E. coli* FASTQ file, the minimum number of queries raises to 44, and to 102 for the gzipped version of that same file. Nonetheless, these numbers are substantially smaller than the usual numbers of required queries, and still reinforce the interest of LRez.

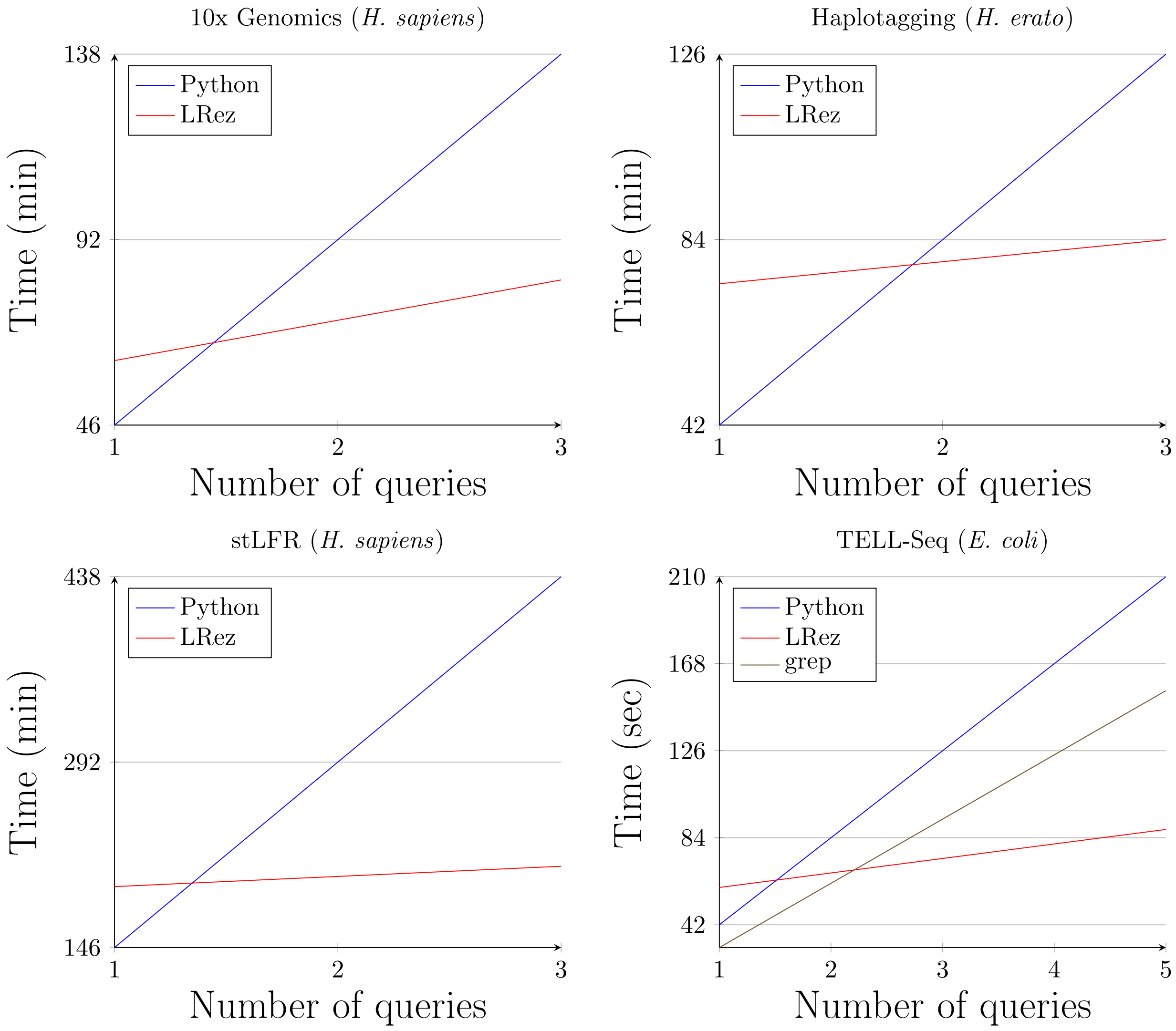

Figure S2: Runtime depending on the number of queries, when querying with a list of 1,000 barcodes, on BAM files. The naive approach based on grep is not represented for the 10x Genomics, Haplotagging and stLFR datasets due to its large runtimes.

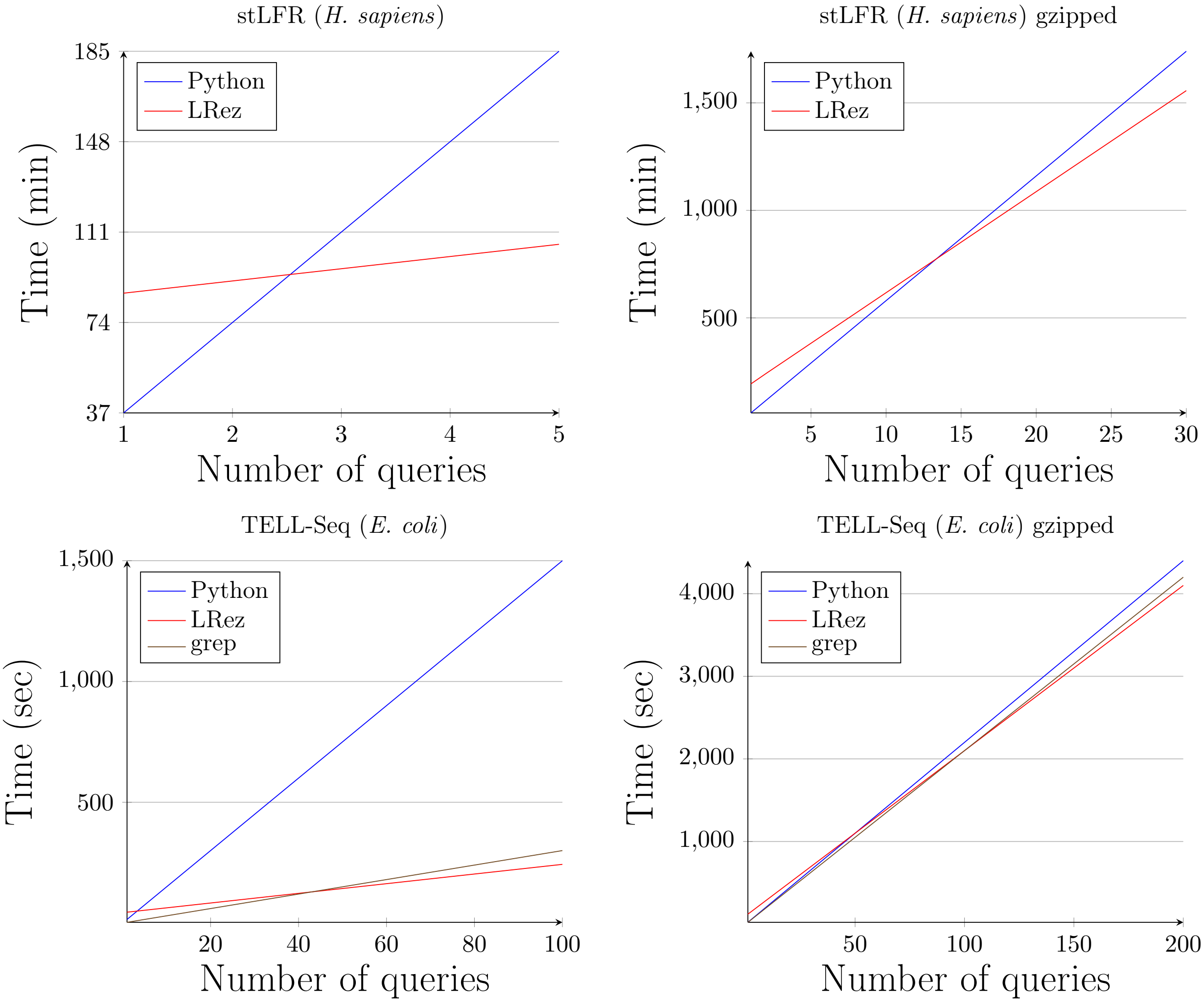

Figure S3: Runtime depending on the number of queries, when querying with a list of 1,000 barcodes, on FASTQ and gzipped FASTQ files. The naive approach based on grep is not represented for the stLFR dataset due to its large runtimes.

6 Running other LRez subcommands

6.1 LRez extract

`LRez extract` allow to extract the barcodes from a given region of a BAM file. This subcommand does not require indexing of the barcodes, and can be run as so:

```
LRez extract --bam bamFile.bam --region chromosome:startPosition-endPosition
```

Additionally, the `-d` switch can be used to include duplicate barcodes. The `LRez extract` subcommand can also be used to extract all the barcodes from a BAM file, using the `-a` switch, as so:

```
LRez extract -b bamFile.bam -a
```

6.2 LRez compare

`LRez compare` allows to compare the number of common barcodes, either between all possible pairs of a specified list of regions, or between a given contig end and all other contigs' ends.

To compute the number of common barcodes between all possible pairs of a given list of regions, the regions of interest must be stored in a file, with one region per line, as so:

```
chr1:1000-2000
chr1:5000-6000
chr2:10000-11000
...
chr15:3000-4000
```

Let's call the previously described file `regions`. `LRez compare` can then be run with the following command:

```
LRez compare -b bamFile.bam -r regions
```

To compute the number of common barcodes between a given contig end and all other contigs' ends, the barcode index of the BAM file must be built, as previously described. `LRez compare` can then be run with the following command, where the `-s` parameter describes the size of the contigs' ends to consider:

```
LRez compare -b bamFile.bam -i bamFile.bci -c contig56 -s 1000
```

7 Example of API usage

The following example illustrates how to build the index from a BAM file, and how to use this index to compute the number of common barcodes between the ends of a given contig, and the ends of all other contigs:

```
bool onlyIndexPrimary = false;
unsigned minQuality = 0;
BarcodesOffsetsIndex index;
index = indexBarcodesOffsetsFromBam(bamFile,
        onlyIndexPrimary, minQuality);
unsigned extSize = 1000;
compareContig(bamFile, index, "chr12", extSize);
```

Other examples of API usage, covering are available on the wiki page of the GitHub repository, at `https://github.com/morispi/LRez/wiki`

References

[1] Zhoutao Chen et al. Ultra-low input single tube linked-read library method enables short-read second-generation sequencing systems to generate highly accurate and economical long-range sequencing information routinely. *Genome Research*, 2020.

[2] Joana I. Meier, Patricio A. Salazar, Marek Kučka, Robert William Davies, Andreea Dréau, Ismael Aldás, Olivia Box Power, Nicola J. Nadeau, Jon R. Bridle, Campbell Rolian, Nicholas H. Barton, W. Owen McMillan, Chris D. Jiggins, and Yingguang Frank Chan. Haplotype tagging reveals parallel formation of hybrid races in two butterfly species. *Proceedings of the National Academy of Sciences*, 118(25):e2015005118, June 2021.
